# Solid-State Nuclear Magnetic Resonance (NMR) and Nuclear Magnetic Relaxation Time Analyses of Molecular Mobility and Compatibility of Plasticized Polyhydroxyalkanoates (PHA) Copolymers

**DOI:** 10.3390/polym10050506

**Published:** 2018-05-07

**Authors:** Masakazu Nishida, Tomoko Tanaka, Yoshio Hayakawa, Masahiro Nishida

**Affiliations:** 1 National Institute of Advanced Industrial Science and Technology (AIST), Shimoshidami 2266-98, Moriyama-ku, Nagoya 463-8560, Japan; tomo.tanaka@aist.go.jp (T.T.); hayakawa-y@aist.go.jp (Y.H.); 2 Nagoya Institute of Technology, Gokiso-cho, Showa-ku, Nagoya, Aichi 466-8555, Japan; nishida.masahiro@nitech.ac.jp

**Keywords:** inedible biomass, polyester, copolymerization, plasticization, solid-state nuclear magnetic resonance (NMR), nuclear magnetic relaxation, variable temperature measurement

## Abstract

The molecular mobility and compatibility of plasticized polyhydroxyalkanoates (PHA) were investigated, focusing on changes due to copolymerization using either flexible poly (butylene succinate) (PBS) or rigid poly(lactic acid) (PLA) units. For the case of a poly(3-hydroxybutyrate) (PHB) unit in plasticized PHA, copolymerization of either PBS or PLA decreased ^1^H and ^13^C spin-lattice relaxation times in the laboratory frame (*T*_1_H and *T*_1_C) in the same manner, while PBS produced a lower ^1^H spin-lattice relaxation time in the rotating frame (*T*_1__ρ_H) than PLA. Both the signals of ^1^H MAS (magic-angle spinning) and ^13^C PST (pulse saturation transfer) MAS nuclear magnetic resonance (NMR) spectra were sharpened and increased by copolymerization with PBS. A variable temperature relaxation time analysis showed that the decrease of *T*_1_H values was dominated by the ^1^H spin diffusion via the interface between PHB and the added polyester because of the good compatibility. Meanwhile, the decrease of *T*_1_C values was dominated by increasingly rapid molecular motions of PHB because of the lowered crystallinity due to the plasticization. Slow molecular motions (kHz order) were enhanced more by the addition of PBS than PLA, although rapid molecular motions (MHz order) were enhanced by either polyester. Several NMR parameters were beneficial for analyzing the manufacturing process as the indexes of polymer compatibility and molecular motions.

## 1. Introduction

For the development of a post-petroleum society, recycling resources from biomass is a high priority. Poly(lactic acid) (PLA) is the most popular biomass-based polymer and is used for daily necessities; however, there is a problem of food security because of the use of edible starch as a main raw material. As one solution to this problem, polyhydroxyalkanoates (PHA) have attracted attention as a biomass-based polymer made from non-edible materials. PHA is the generic name for a biopolyester family, consisting of the following polymers, poly(3-hydroxybutylate) (PHB) and poly(3-hydroxyvalerate) (PHV), including their isomeric polymers and co-polymers; the characteristic properties of PHA resulting from the chain length and composition could be tuned to various industrial applications [1]. Microbial production is the most commonly used method for supplying PHA; not only carbohydrates but also fatty acids were used as the carbon source depending on the bacterial strain employed [2,3,4]. For mechanical applications, crystal structures and crystallization kinetics have been studied in connection with biosynthesis and the polymerization mechanism [5]. Furthermore, PHA is expected to be useful in the medical field because of its biodegradability and biocompatibility; the improvement of solubility of PHA in water was studied by modification of the polymer chain [6]. However, PHA has limitations in its thermal and physical properties, such as low processability, brittleness, hydrophilicity and inferior biocompatibility; plasticizers and plasticization processes were studied to resolve these limitations [7].

In order to improve the processability and brittleness of PHA, modifications of PHB and PHB-based polymers have been intensively studied as follows. Modification of PHB with plasticizers and other copolymers as the improvement method was analyzed with differential scanning calorimetry (DSC), thermogravimetric analysis (TG), and a melt flow indexer [8]. Biobased epoxidized broccoli oil can plasticize and stabilize PHA and its plasticization process was characterized by several instrumental analyses [9]. Copolymerization and blending for PHB and PHB-based polymers is a promising method for improving their material properties. For example, the material properties and processing options of PHB was improved using reactive melt processing of PHB and PLA via peroxide-induced cross-linking [10]. Chemical compatibilization using dicumyl peroxide could also ameliorate the mechanical properties, such as tensile strength, impact toughness, and elongation at break, of blends of PHB and PHB–PHV copolymers with poly(butylene succinate) (PBS) [11]. Impact properties were improved by melt extrusion and subsequent injection molding of a PHB/poly(ε-caprolactone) (PCL) blend, which was characterized by DSC, TG, and field emission scanning electron microscopy (FESEM) [12]. Injection molding could also provide biocomposites based on PHB/PHV copolymers and lignocellulosic filler together with blow extrusion [13]. For packaging applications, blends of PHB/PHV copolymer and PLA were studied, focusing on their mechanical and barrier properties [14]. Tensile properties of PHA blends were significantly affected by their PCL content; the mechanism of interaction was analyzed by rheo-optical near-infrared (NIR) spectroscopy [15].

Solid-state nuclear magnetic resonance (NMR) is an extremely useful analytical method to obtain information about chemical behaviors and molecular dynamics. The molecular dynamics of amorphous and crystalline PHB were analyzed with ^13^C magic-angle spinning (MAS) NMR and ^13^C spin-lattice relaxation times (*T*_1_C) [16]. A solid-state NMR study of bacterial PHB-based copolyesters was performed using three ^13^C MAS NMR methods, cross-polarization (CP), dipolar decoupling (DD), and pulse saturation transfer (PST), as well as variable contact times in ^13^C CP-MAS NMR [17]. Semi-crystalline PHB and PHB–PHV copolymer were studied by several solid-state NMR spectral and relaxation time analyses to reveal the domain structure and mobility [18]. Reinforcement of PHA with cross-linked cellulose acetate was monitored by solid-state ^13^C NMR [19]. Time-domain nuclear magnetic resonance (TD-NMR) was also useful for determining the morphology of PHB/PCL blends [20]. Very recently, plasticized PLA/PHA polymer blends were studied by single-pulse NMR and *T*_1_C [21].

As an example of the use of solid-state NMR for analyzing the mechanical properties of biomass-based polymers, we have already studied PLA fibers produced by an extrusion process to reveal the correlation between tensile properties and spin-lattice relaxation time in PLA/PCL copolymer fibers [22], the morphological changes of PLA/organic composite fibers [23], and the effects of nucleating and plasticization in drawn PLA fibers during degradation [24]. In addition, using relaxation time analyses as a possible method of quality control of biomass-based materials, we have also revealed the interaction of water molecules with phenol formaldehyde resin-impregnated soft wood [25], that with biomass constituent in Japanese cypress [26], and that with superheated steam-treated plant materials [27]. On the basis of our previous solid-state NMR and relaxation time studies of the biomass-based materials, the present study is intended to clarify the information necessary for improvements to the processability and brittleness of PHA. At first, focusing on two kinds of plasticized PHA copolymers including PBS and PLA, we measure not only their ^13^C CP-MAS and ^13^C PST-MAS NMR spectra but also three spin-lattice relaxation times, *T*_1_H, *T*_1_C, and *T*_1__ρ_H, at ambient temperature to show the effects on these spectra and parameters of these added polyesters. Next, by combining the results at ambient temperature with additional ^1^H MAS NMR, *T*_1_H, and *T*_1_C measurements at variable temperatures, we will reveal how the modification of the added polyesters affects the molecular mobility and the chemical compatibility between the polymer constituents.

## 2. Materials and Methods

### 2.1. Materials

Pre-plasticized PHB pellets were purchased from Biomer, Krailling, Germany. PBS pellets were purchased from Showa Highpolymer Co. Ltd., Tokyo, Japan. PLA pellets were purchased from Mitsui Chemical Co. Ltd., Tokyo, Japan. The following two kinds of plasticized PHA pellet used for the present study were purchased from G5 Manufacturing, Singapore. The PHA4422P pellet consisted of 65% PHA, 30% PBS, and 5% crosslinking reagent and the catalog specification values of PHA4422P were as follows: melt flow rate: 2.5 g/10 min; tensile strength: 21 MPa; elongation: 600%; Izod impact strength: ≥30 kJ/m^2^; flexural modulus: 600 MPa. The PHA5533L pellet consisted of 60% PHA, 35% PLA, and 5% crosslinking reagent and the catalog specification values of PHA5533L were as follows: melt flow rate: 4.0 g/10 min; tensile strength: 18 MPa; elongation: 100%; Izod impact strength: 22 kJ/m^2^; flexural modulus: 1000 MPa. All materials were used without further purification.

### 2.2. Differential Scanning Calorimetry (DSC)

Differential scanning calorimetry (DSC) was performed on a DSC-60 instrument (Shimadzu Co. Ltd., Kyoto, Japan). The sample (30 mg) was sealed in an aluminum pan and then was heated from 30 to 250 °C at a heating rate of 10 °C/min under 250 mL/min N_2_ flow. An empty aluminum pas was used as a reference.

### 2.3. X-ray Diffraction (XRD)

The crushed sample was placed on a sample holder and then the X-ray diffraction (XRD) under Cu Kα irradiation was measured on a RINT-Ultima II diffratometer (Rigaku Co. Ltd., Tokyo, Japan). The diffratometer was operating at 40 kV and 40 mA and the diffractogram was recorded at 2θ angles between 5° and 40°.

### 2.4. Nuclear Magnetic Resonance (NMR) Measurements at Ambient Temperature

Solution nuclear magnetic resonance (NMR) spectra were measured on a Varian INOVA-300 (Palo Alto, CA, USA) with a Varian 5 mm four-nucleus auto-NMR triple-resonance liquid probe in CDCl_3_ as a solvent. ^1^H NMR spectra were collected at 299.95 MHz with a 7.0 μs π/2 pulse for the ^1^H nuclei and 3.74 s acquisition period over a 4.5 kHz spectra width with 1.2 s recycle delay in 16–128 transients. ^13^C NMR spectra were collected at 75.43 MHz with a 6.5 μs π/2 pulse for the ^13^C nuclei and 1.82 s acquisition period over a 16.5 kHz spectra width with 1.19 s recycle delay in 2048–15,000 transients.

Solid-state NMR spectra were measured on a Varian 400 NMR system spectrometer (Palo Alto, CA, USA) with a Varian 4 mm double-resonance T3 solid probe. The samples were placed in a 4 mm ZrO_2_ rotor spun at 15 kHz over a temperature range of 20–22 °C. Solid-state ^13^C MAS NMR spectra were measured at 100.56 MHz for the ^13^C nuclei and were collected with a 40 ms acquisition period over a 30.7 kHz spectral width in 1024–2048 transients. Proton decoupling was performed with an 86 kHz ^1^H decoupling radio frequency with a small phase incremental alteration (SPINAL) decoupling pulse sequence. The cross polarization and magic angle spinning (CP-MAS) NMR spectra were measured with a 5.0 s recycle delay using a ramped-amplitude pulse sequence with a 2 ms contact time and a 2.5 μs π/2 pulse for the ^1^H nuclei. The amplitude of the ^1^H nuclei was ramped up linearly from 90.5% of its final value during the cross-polarization contact time. The pulse saturation transfer and magic angle spinning (PST-MAS) NMR spectra were measured with a 2.6 μs π/2 pulse for the ^13^C nuclei with a 5 s recycle delay after saturation of ^1^H nuclei with 13 consecutive 2.5 μs pulses and a 27.5 μs delay.

### 2.5. Nuclear Magnetic Relaxation Time Analyses

The ^1^H spin-lattice relaxation time in the laboratory frame (*T*_1_H) was indirectly measured via detection of ^13^C resonance enhanced by cross-polarization, applied after a π pulse to ^1^H nuclei with the inversion recovery method. The ^13^C spin-lattice relaxation time in the laboratory frame (*T*_1_C) was measured with the conventional Torchia’s pulse sequence [28]. The ^1^H spin-lattice relaxation time in the rotating frame (*T*_1__ρ_H) was indirectly measured via detection of ^13^C resonance enhanced by cross-polarization applied after a π/2–τ–spin lock pulse (86 kHz of radio frequency) with variable spin lock times over a range of 100–8000 µs. The relaxation time analyses were performed with the same solid-state probe with the same contact time and acquisition period used for the ^13^C CP-MAS NMR spectrum.

### 2.6. Variable Temperature Solid-State NMR and Relaxation Time Measurements

Variable temperature solid-state NMR spectra and relaxation times were also measured on the same spectrometer and probe as the ambient temperature measurements. Low-temperature measurement was started at a low temperature side of −40 °C and the temperature increased to 10 °C. High-temperature measurement was started at a low temperature side of 30 °C and heating to 50 °C. One step of heating consisted of 10 °C (^1^H MAS NMR, *T*_1_H, *T*_1_C high temperature) or 20 °C (*T*_1_C low temperature). The samples were placed in a 4 mm ZrO_2_ rotor spun at 12 kHz (low temperature) and 15 kHz (high temperature) under nitrogen flow. Low temperature ^1^H MAS NMR spectra were measured at 399.88 MHz for the ^1^H nuclei and were collected with a 2.9 μs π/2 pulse for the ^1^H nuclei and 20 ms acquisition period over a 30.5 kHz spectral width in 4 transients, and a 3 s recycle delay. Low and high temperature *T*_1_H and *T*_1_C were measured using the same acquisition parameters as described above. Each low and high temperature measurement was performed independently because it was difficult to control low temperature for a long time due to the use for liquid nitrogen.

## 3. Results and Discussion

### 3.1. Polymer Properties of Plasticized Polyhydroxyalkanoates (PHA) Pellets

The pre-plasticized PHB pellet showed poor processability because of stiffness and low solubility. Copolymerization with the biodegradable polyesters, PBS and PLA, improved the mechanical properties of PHA. According to the catalog specification values, copolymerization with PBS increased elongation and Izod impact strength, while copolymerization with PLA increased melt flow rate and flexural modulus. Actually, when installing a sample into the solid-state NMR rotor, the pre-plasticized PHB was difficult to cut and/or crush, even though the plasticized PHA copolymers were easily deformable to prepare a solid-state NMR sample.

**Figure 1:**
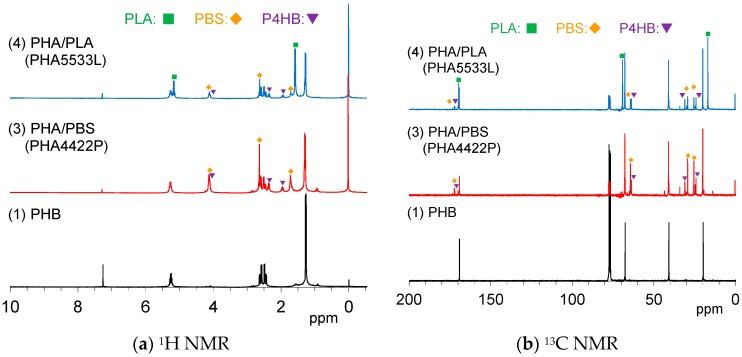
Solution nuclear magnetic resonance (NMR) spectra of plasticized polyhydroxyalkanoates (PHA) copolymers and original poly(3-hydroxybutyrate) (PHB) in CDCl_3_.

In order to determine in detail the constituents of the plasticized PHA pellets connected with the mechanical properties, solution NMR spectra were measured in CDCl_3_. Figure 1a shows ^1^H NMR spectra of the pre-plasticized PHB and the plasticized PHA pellets. The PHA4422P pellet (3) showed three PHB signals, doublet (CH_3_, 1.28 ppm), quartet-doublet (CH_2_, 5.24 ppm), and quartet (CH, 5.26 ppm), which can be also observed for the original PHB (1). PHA4422P also had small amount of poly(4-hydrobutyrate) (P4HB) [29], and the added PBS for the copolymerization. One can see a trace peak at 0.98 ppm, in which region a CH_3_ group of PHA having long alkyl groups might appear; however, the actual structure could not be assigned at this time. Meanwhile, ^1^H NMR spectra also indicated that the PHA5533L pellet (4) consisted of PHB, the added PLA, and a small amount of PBS and P4HB. Minor constituents in each pellet could be confirmed by ^13^C NMR spectra (Figure 1b). The signals of P4HB in both PHA4422P (3) and PHA5533L (4) also appeared not only in the aliphatic region but also in the carbonyl region.

**Figure 2:**
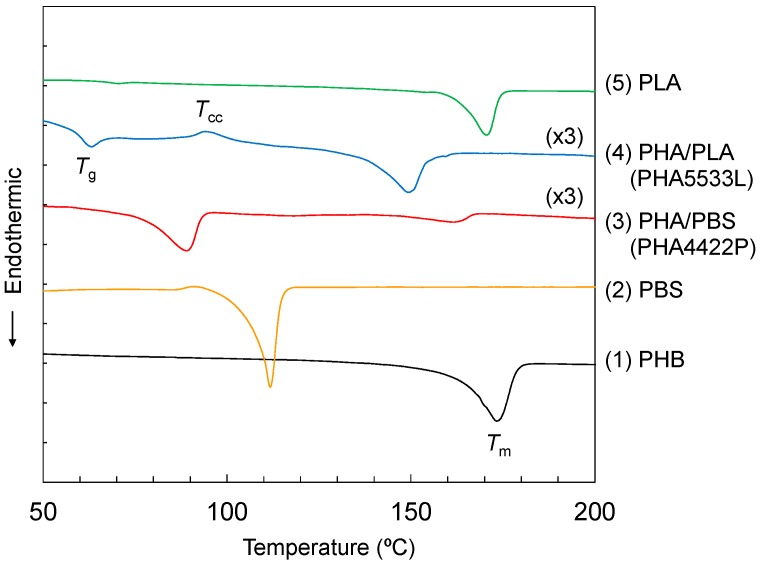
Differential scanning calorimetry (DSC) heating curves of plasticized PHA copolymers and constituent polymers.

The improvement of mechanical properties due to the copolymerization with the biodegradable polyesters could be explained by a change of thermal characteristics. Figure 2 shows the DSC curves for the modified PHA pellets and the reference polymers. The melting peak (*T*_m_) of the original PHB (1) appeared at a relatively high temperature (174 °C) with no other endothermic and exothermic peaks. Of the constituent polyesters used for the copolymerization, PBS (2) had a lower *T*_m_ (112 °C) while PLA (5) also had a high *T*_m_ (171 °C). Interestingly, the copolymerization resulted in a decrease of *T*_m_ to a value lower than the initial *T*_m_ of the constituent polyesters, although the heat capacities of both plasticized PHA pellets were smaller than the original PHB. The PHA4422P pellet (3) had a lower *T*_m_ (89 °C) with higher temperature small endothermic peaks. In contrast, the PHA5533L pellet (4) had a glass transition peak with relaxation (*T*_g_: 64 °C) and cold crystallization (*T*_cc_: 95 °C), and a quite high *T*_m_ (150 °C). Considering the DSC results together with the solution NMR, small amounts of P4HB and PBS were extremely effective for changing the thermal properties of the PHA5533L pellet. Moreover, the DSC results showed that the plasticization of PHA improved processability because of the decrease of *T*_m_ (PBS) or *T*_g_ (PLA).

**Figure 3:**
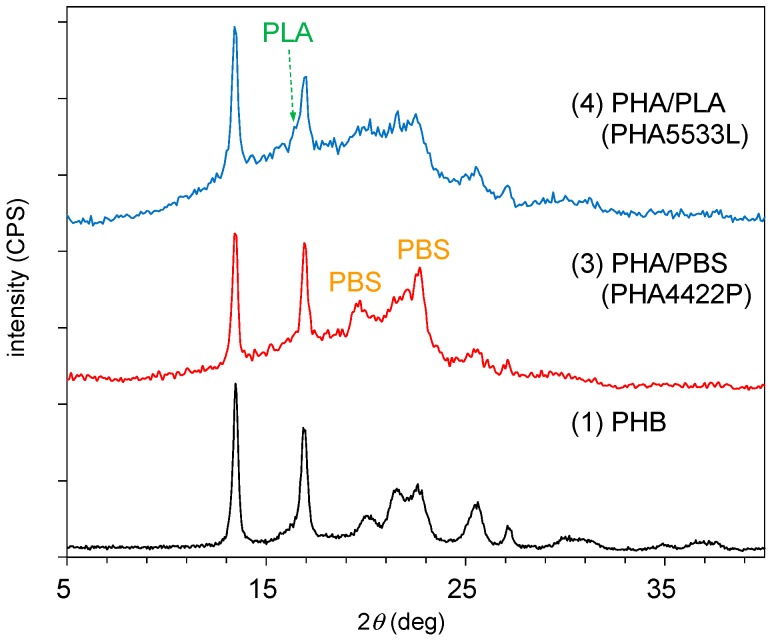
X-ray diffraction (XRD) patterns of plasticized PHA copolymers and original PHB.

The copolymerization with the biodegradable polyesters also changed the crystallinity of the PHA. Figure 3 shows changes in the XRD patterns of PHA by the addition of other polyesters. The original PHB (1) had moderate crystallinity, showing orthorhombic crystal planes with major sharp peaks at 2θ = 13.5° (020) and 16.9° (110). After the copolymerization of PHA with PBS [PHA4422P (3)], new peaks that originated from PBS appeared at 2θ = 19.6° (020) and 22.6° (110) overlapped with the (021) and (111) peaks of PBS [30]. At the same time, the amorphous peak of PHA clearly increased. The copolymerization of PHA with PLA [PHA5533L (4)] increased further the amorphous peak of PHA; only a small peak of PLA was observed at 2θ = 16.4° (100) [24]. In both cases of PHA4422P and PHA5533L, characteristic XRD peaks from P4HB could not be assigned. The XRD results showed that the copolymerization of both biodegradable polymers decreased the crystallinity of PHA and that the decrease was more pronounced for the copolymerization with PLA than with PBS.

### 3.2. ^13^C Magic-Angle Spinning (MAS) NMR of Plasticized PHA Copolymers and Constituent Polymers

**Figure 4:**
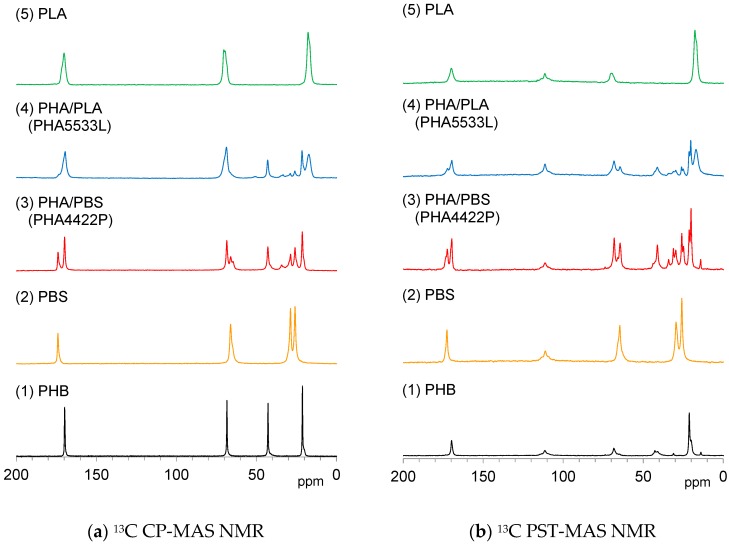
^13^C Magic-angle spinning (MAS) NMR spectra of plasticized PHA copolymers and constituent polymers.

Solid-state ^13^C NMR is a powerful tool for analyzing the crystallinity and mobility of polymer chains. The ^13^C CP-MAS NMR variant is commonly used in polymer science; it utilizes the magnetization transfer from ^1^H nuclei to ^13^C nuclei, enhancing the signal of a rigid substituent. Figure 4a shows ^13^C CP-MAS NMR spectra of plasticized PHA copolymers and constituent polymers. Each signal for the polymer constituents, PHB (1), PBS (2) and PLA (5) could be effectively detected in the ^13^C CP-MAS NMR spectra regardless of the flexibility and the rigidity of each polymer. The ^13^C CP-MAS signals of the plasticized PHA4422P (3) could be assigned as PHB and PBS, while those of the plasticized PHA5533L (4) as PHB, PLA, and PBS. The isomeric polymer P4HB could be also detected in both PHA4422P (3) and PHA5533L (4).

Meanwhile, ^13^C PST-MAS NMR is a method utilizing the nuclear Overhauser effect, resulting in the enhancement of a signal of a substituent having high mobility near a hydrogen atom [27]. Figure 4b shows ^13^C PST-MAS NMR spectra of plasticized PHA copolymers and constituent polymers. Actually, the CH_3_ group of PLA (5) and the OCH_2_*C*H_2_ group of PBS (2) showed larger intensities than other substituents because the amplitudes of their molecular motions were higher. For the pre-plasticized PHB (1), the CH_3_ group had a high signal intensity compared to the other signals. Interestingly, the copolymerization of PHA with PBS [PHA4422P (3)] increased the signal from CH_2_ and CH_3_ groups accompanied by the appearance of signals from the minor isomeric polymer P4HB. In both PHA (1) and PHA4422P (3), trace signals appeared at the higher field near the CH_3_ groups’ signal. These small signals originated from trace amounts of PHA having long alkyl groups. In contrast, the copolymerization of PHA with PLA [PHA5533L (4)] did not increase the CH_2_ and CH_3_ groups’ signals but only gave the sum of the signals of PHA, P4HB, PLA, and PBS. The results of the ^13^C PST-MAS NMR spectra confirmed that PBS enhanced molecular mobility of the whole copolymer system. Next, we consider the different trends in molecular mobility between the plasticization with PBS [PHA4422P (3)] and PLA [PHA5533L (4)] revealed by their spin-lattice relaxation times.

### 3.3. Spin-Lattice Relaxation Times in the Laboratory Frame of Plasticized PHA Copolymers and Constituent Polymers

The ^1^H spin-lattice relaxation time in the laboratory frame (*T*_1_H) is reduced by rapid molecular motions matched to the Larmor frequency. For example, the ^1^H spin-lattice relaxation in the laboratory frame (*T*_1_H relaxation) of PLA is promoted by the rotation of a CH_3_ group around its *C*_3_ axis. Moreover, the *T*_1_H value of each substituent in the same polymer chain adopts an averaged value because of ^1^H spin diffusion.

**Figure 5:**
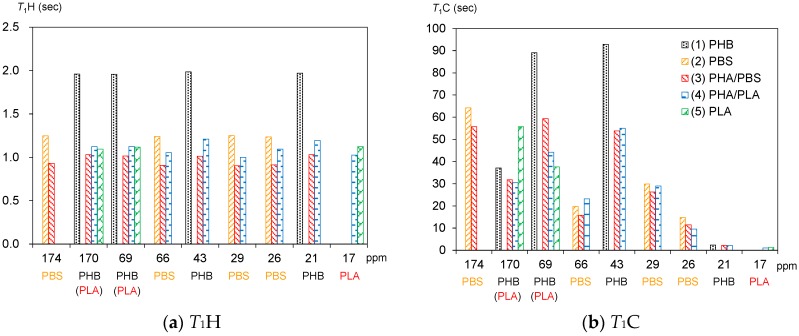
Changes of *T*_1_H and *T*_1_C values with copolymerization. The horizontal axis shows the chemical shift of each signal appearing in the ^13^C cross-polarization (CP) MAS NMR spectra while the vertical axis shows the corresponding *T*_1_H (**a**) and *T*_1_C (**b**) values.

Figure 5a shows *T*_1_H values of plasticized PHA copolymers and constituent polymers. As described in the above paragraph, the *T*_1_H values of PLA (5) were averaged by the spin diffusion to have almost the same values (1.1 s) due to the *T*_1_H relaxation via the rotation of the CH_3_ group. Although the *T*_1_H values of PBS (2) also showed averaged values (1.2 s) similar to PLA, the *T*_1_H relaxation of PBS cannot be explained directly by molecular motions of a specific substituent, such as a CH_3_ group. Moreover, the *T*_1_H values of PHB (1) had longer averaged values (2.0 s) even though the *T*_1_H relaxation occurred via the motion of CH_3_ group. The different *T*_1_H values of PLA (5) and PHB (1) having the same *T*_1_H relaxation route were due to whether the frequency of CH_3_ motion matched the Larmor frequency, or not.

Copolymerization with the biodegradable polyesters significantly decreased the *T*_1_H values of PHB. For the plasticized PHA4422P (3) in particular, the addition of PBS decreased not only the *T*_1_H values of the PHB unit (1.0 s) but also the *T*_1_H values of the PBS unit (0.9 s). Meanwhile, for the plasticized PHA5533L (4), the addition of PLA also decreased the *T*_1_H values of the PHB unit (1.2 s), although the *T*_1_H values of the PLA unit kept the original values of the PLA pellet (1.05 s). At the same time, the *T*_1_H values of trace amounts of the PBS unit slightly decreased (1.0 s) in PHA5533L. These *T*_1_H reductions were caused by the enhancement of the *T*_1_H relaxation due to the heterogeneity that arose from the amorphous moiety, as we have previously described for PLA copolymer fibers [24].

The ^13^C spin-lattice relaxation time in the laboratory frame (*T*_1_C) can provide information on molecular motions for each substituent more directly because it excludes the effect of ^1^H spin diffusion. Figure 5b shows *T*_1_C values of plasticized PHA copolymers and constituent polymers. One can see a different *T*_1_C value for each substituent in the pre-plasticized PHB (1): C=O, 37 s; CH, 89 s; CH_2_, 92 s; CH_3_, 2.4 s. The *T*_1_C value of CH_3_ nearly equaled the *T*_1_H (2.0 s) value because the ^13^C spin-lattice relaxation (*T*_1_C relaxation) was also promoted by the CH_3_ rotation, the same as for the *T*_1_H relaxation. This closeness between the *T*_1_C relaxation of CH_3_ and the *T*_1_H due to the CH_3_ rotation also arose in PLA (5) (*T*_1_C, 1.4 s; *T*_1_H, 1.1); however, the C=O group (56 s) showed a longer value than the CH group (38 s) in PLA, unlike the pre-plasticized PHB. One can also see the longer *T*_1_C value of the C=O group (64 s) and moderate *T*_1_C values of CH_2_ groups (15–30 s) in PBS (2). Although PBS had high flexibility, the molecular motion of each substituent did not match with the Larmor frequency of the ^13^C nuclei.

The reduction of *T*_1_C values due to the copolymerization was also observed not only in PHA4422P (3) but also in PHA5533L (4), similarly to the *T*_1_H values. The *T*_1_C reduction of the PHB unit was significant for C(=O) CH_2_ [original 89 s; 4422(PBS), 59 s; 5533(PLA), 44 s] and OCH_2_ [original, 93 s; 4422(PBS), 54 s; 5533(PLA), 55 s]. In contrast, the *T*_1_C value of the C=O of the PBS unit changed less [original, 37 s; 4422(PBS), 30 s; 5533(PLA), 32 s] as well as the *T*_1_C of CH_3_ [original, 2.4 s; 4422(PBS), 2.2 s; 5533(PLA), 2.1 s]. Moreover, the *T*_1_C values of the PBS unit in PHA4422P (3) and the *T*_1_C values of the PLA unit CH_3_ in PHA5533L (4) were slightly reduced by the copolymerization, compared with the original *T*_1_C values. Since the copolymerization with either PBS or PLA decreased the crystallinity of the PHB unit (Figure 3), the reduction of the *T*_1_C values of the PHB unit could be also explained by a change of *T*_1_C relaxation due to the heterogeneity arising from the amorphous moiety. We will discuss the relationship of the heterogeneity with the compatibility of the copolymer components and molecular mobility later.

### 3.4. Spin-Lattice Relaxation Time in the Rotating Frame of Plasticized PHA Copolymers and Constituent Polymers

The ^1^H spin-lattice relaxation in the rotating frame (*T*_1__ρ_H relaxation) is promoted by molecular motions near the spin-locking frequency, which is much less than the Larmor frequency. That is, the ^1^H spin-lattice relaxation time in the rotating frame (*T*_1__ρ_H) is affected more than the *T*_1_H value by slower molecular motions, such as the main chain motion of polymers.

**Figure 6:**
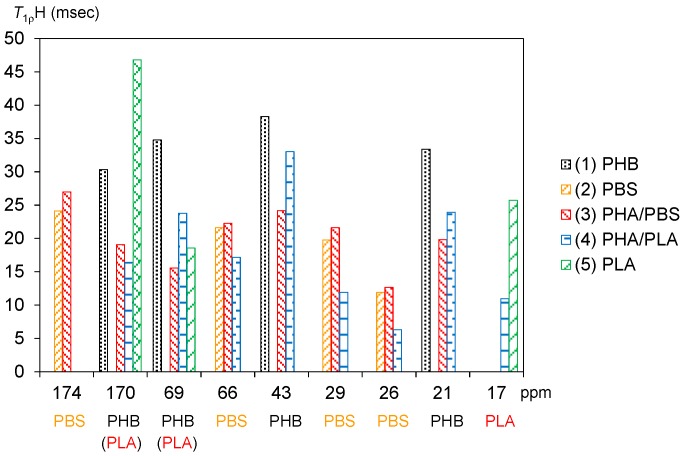
Changes of *T*_1__ρ_H values with copolymerization. The horizontal axis shows the chemical shift of each signal appearing in the ^13^C CP-MAS NMR spectra while the vertical axis shows the corresponding *T*_1__ρ_H value.

Figure 6 shows *T*_1__ρ_H values of plasticized PHA copolymers and constituent polymers. The significant decreases of the *T*_1_H values for PHB due to the copolymerization with PBS and PLA were also observed for the *T*_1__ρ_H values. The original PHB (1) showed comparable *T*_1__ρ_H values for each substituent as follows: C=O, 30 ms; CH, 35 ms; CH_2_, 38 ms; CH_3_, 33 ms. The *T*_1__ρ_H values of the PHB unit were obviously reduced by the copolymerization with the biodegradable polyesters. Except for the C=O group, the reduction ratios of *T*_1__ρ_H values were larger in the copolymer with PBS [PHA4422P (3): 16–24 ms] than in the copolymer with PLA [PHA5533L (4): 24–33 ms]. Meanwhile, PBS (2) took lower *T*_1__ρ_H values, such as C=O (24 ms), OCH_2_ (22 ms), C(=O) CH_2_ (20 ms), CH_2_ (12 ms). The *T*_1__ρ_H values of PBS unit stayed almost unchanged for PHA4422P (3) (13–27 ms) while they decreased in PHA5533L (4) (6–17 ms). The *T*_1__ρ_H values of CH_3_ in PLA (5) also decreased with the copolymerization (PLA, 26 ms; PHA5533L, 11 ms).

As shown above, since the spin-lattice relaxation times in the laboratory frame (*T*_1_H and *T*_1_C) showed a similar trend between PHA4422P (PBS) and PHA5533L (PLA), rapid molecular motions (MHz) matched with the Larmor frequency in both plasticized PHA copolymers was enhanced by the plasticization. On the other hand, the spin-lattice relaxation time in the rotating frame (*T*_1__ρ_H) decreased more in PHA4422P. Therefore, the plasticization with the flexible PBS enhanced slow molecular motions (kHz) corresponding to the spin-locking frequency more than did PLA. We previously discussed the correlation of increase of the PST-MAS signals and *T*_1__ρ_H values for phenol formaldehyde resin-impregnated soft wood [25]. Similar to this previous result, the increases of the ^13^C PST-MAS NMR signals here could be explained by the enhancement of slow molecular motions (kHz) due to the plasticization with PBS. We will discuss the increase of ^1^H MAS NMR signals related to the slow molecular motion in the next subsection.

### 3.5. Changes of ^1^H MAS NMR with Rising Temperature

**Figure 7:**
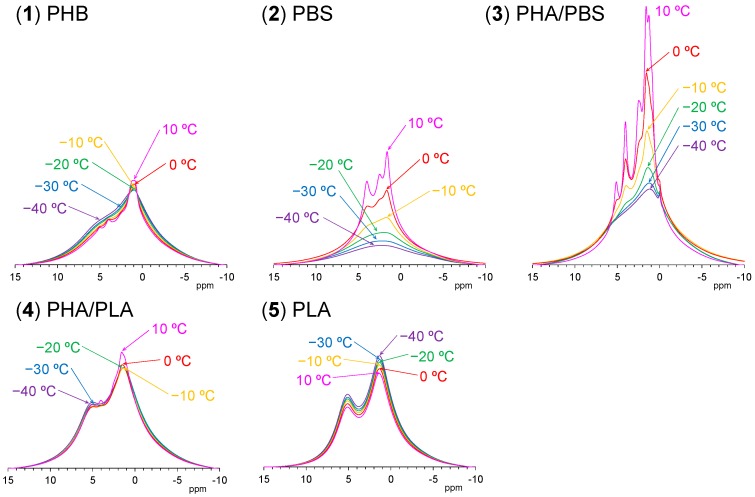
Variable temperature ^1^H MAS NMR spectra of plasticized PHA copolymers and constituent polymers. The temperature was increased from 40 °C to −10 °C.

Figure 7 shows variable temperature ^1^H MAS NMR spectra of plasticized PHA copolymers and constituent polymers. Because of strong dipolar–dipolar interactions of ^1^H nuclei with each other, the ^1^H signals in the solid-state NMR appear as broad overlapped signals. The original PHB (1) showed only such signals, which changed little with increasing temperature; the CH_3_ signal sharpened and increased and the broader peaks in the CH and CH_2_ regions decreased at the same time. This showed that the molecular mobility of PHB (1) alone remained almost unchanged by the temperature rise. For the flexible PBS (2), however, every signal significantly sharpened and increased with increasing temperature, even though only very broad and weak peaks appeared at low temperatures (−40 °C). That is, the mobility of flexible PBS (2) was greatly affected by the temperature rise, resulting in a sharpened and larger ^1^H MAS signals. Interestingly, the copolymerization of PHA with PBS [PHA4422P (3)] enlarged and sharpened the signals of not only the PBS unit but also the PHB unit. The rate of increase of the ^1^H signals for both PHA and PBS units in PHA4422P (3) was greater than for PBS (2) alone; thus, the copolymerization of PHA with PBS increased the mobility of not only the PHA unit but also the PBS unit compared with the original polymers. Meanwhile, the ^1^H signal intensity of rigid PLA (5) decreased with increasing temperature as seen for woody materials [26,27]. These ^1^H signal decreases may occur due to sensitivity change of the detecting coil in the probe; now, we are still searching other reasons related to the magnetic relaxation. The plasticized PHA5533L (4) showed only the overlapped ^1^H signal of PHB and PLA units, which simply changed in the same manner as each polymer alone, with no increase in signal or sharpness. According to the variable ^1^H MAS NMR results, the flexibility of the polymer significantly enhanced slow molecular motions (kHz) corresponding to the *T*_1__ρ_H relaxation, resulting in sharp and large ^1^H signals after plasticization with PBS. The slow molecular motion (kHz order) corresponded to the motion of polymer chains, which was enhanced at temperatures over −10 °C.

### 3.6. Changes of Spin-Lattice Relaxation Times in the Laboratory Frame with Rising Temperature

**Figure 8:**
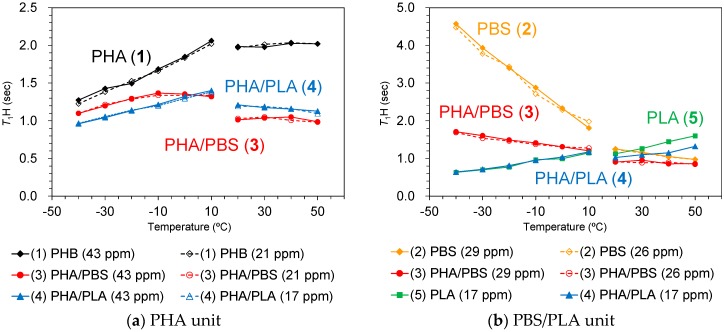
*T*_1_H changes of PHA (**a**) and poly(butylene succinate)/poly(lactic acid) (PBS/PLA) units (**b**) in plasticized PHA copolymers with rising temperature. The temperature was increased from 40 °C to −10 °C (low temperature side) and from 20 °C to 50 °C (high temperature side).

**Figure 9:**
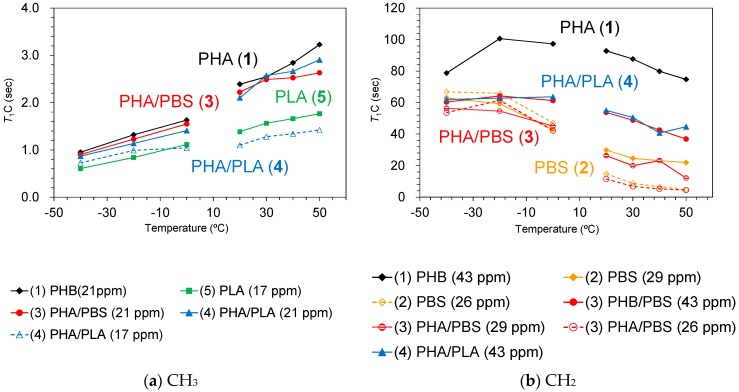
*T*_1_C changes of CH_3_ (**a**) and CH_2_ (**b**) groups in plasticized PHA copolymers with rising temperature. The temperature was increased from 40 °C to 0 °C (low-temperature side) and from 20 °C to 50 °C (high-temperature side).

In order to investigate the rapid molecular motions (MHz) that participated in the spin-lattice relaxation in the laboratory frame, rising temperature *T*_1_H and *T*_1_C measurements were performed in two temperature regions. The *T*_1_H and *T*_1_C changes due to the temperature rises are summarized in Figure 8 and Figure 9, respectively.

The change in trends of *T*_1_H and *T*_1_C values was closely related to the existence of CH_3_ groups. The *T*_1_H values of PHB (1) (Figure 8a, black line) and PLA (5) (Figure 8b, green line), both of which had CH_3_ groups, increased with increasing temperature except for the high temperature region of PHB (1). In contrast, PBS (2) (Figure 8b, orange line), which had no CH_3_ group, decreased with increasing temperature; the rate of decrease was significantly faster in the low-temperature region. The different change trend that correlates with the existence of CH_3_ groups was also observed in the *T*_1_C values. The *T*_1_C values of CH_3_ groups increased monotonically with increasing temperature in both PHB (1) (black line) and PLA (5) (green line) up to 50 °C (Figure 9a). Conversely, a decrease with rising temperature was observed for the *T*_1_C value of each substituent in PBS (Figure 9b, orange line). The CH_2_ group in PHB (43 ppm) has a very long *T*_1_C value (over 80 s), which increased in the low temperature and decreased in the high temperature regions (Figure 9b, black line). These trends of *T*_1_ values showed that the rotation of CH_3_ in PHB and PLA had a lower correlation time (τ_c_) than molecular motions of CH_2_ in PHB and PBS.

Although the *T*_1_H value of the PHA unit in PHA4422P (3) increased to −10 °C, it started to decrease over −10 °C (Figure 8a, red line). The *T*_1_H value of PBS unit in PHA4422P (3) conversely decreased and the rate of decrease became smaller in the high-temperature region (Figure 8b, red line). As a result, the *T*_1_H values of PHA and PBS units in PHA4422P (3) approached each other over −10 °C and these approached the *T*_1_H values of PHA and PBS units which maintained their relationship in the higher temperature region. The approached *T*_1_H values are caused by the ^1^H spin diffusion via the interface between PHB and PBS, which increases with improving polymer compatibility of PHA and PBS units above −10 °C. This interfacial ^1^H spin diffusion possibly enhanced the T_1_H relaxation in the whole system of PHB/PBS copolymer. The improved compatibility for PHA4422P (3) also made ^1^H MAS NMR signals sharper and larger at temperatures over −10 °C (Figure 7). Although a *T*_1_H reduction of the PHA unit was also observed in PHA5533L (4) (Figure 8a, blue line), no inflection points of the *T*_1_H curve were observed in the low-temperature region and the ^1^H MAS NMR spectra changed only slightly in PHA5533L (4). These results indicated that the polymer compatibility between PHA and PLA was less improved in PHA5533L (4) although the *T*_1_H relaxation was enhanced by the heterogeneity.

The *T*_1_C value of the CH_2_ groups in the plasticized PHA copolymers gave information about rapid molecular motions (MHz order) of the polymer chain. In all temperature ranges, the PHB unit of the plasticized PHA copolymers has a low *T*_1_C value, compared with the pre-plasticized PHB, although the PBS units had similar *T*_1_C values regardless of the plasticization. The heterogeneity that had arisen from the amorphous moiety also increased the molecular mobility of the PHB chain, which enhanced the *T*_1_C relaxation to produce the lowered *T*_1_C value of the PHB unit. Meanwhile, the *T*_1_C value of CH_3_ groups in the plasticized PHA copolymers provides information on the local motion of CH_3_ closely related to the *T*_1_H relaxation. The reduction of the *T*_1_C value of CH_3_ groups due to copolymerization was limited, unlike the *T*_1_H value. Thus, the change of *T*_1_H was not caused by the molecular motion of CH_3_ groups, but by the interaction between constituent polymers.

In conclusion, *T*_1_H values can act as an index of polymer compatibility due to plasticization, while *T*_1_C values of CH_2_ groups can provide an index of rapid molecular motions of constituent polymers. Moreover, the *T*_1__ρ_H values can index slow molecular motions of the polymer main chain, along with the ^1^H and ^13^C PST-MAS NMR spectra. Since copolymerization of PHA with PBS improved elongation and impact properties, the increase in slow molecular motions and compatibility of the PHA/PBS copolymer is possibly related to such polymer properties. Next, we are planning to study other biomass-based polymers to relate polymer properties to the indexes in solid-state NMR analytical methods proposed in the present study.

## 4. Conclusions

With the aim of extending the application of solid-state NMR to biomass-based polymers, two kinds of biodegradable polyesters, poly (butylene succinate) (PBS) and poly(lactic acid) (PLA), were examined for the plasticization of the biopolymers of no nutritional use, polyhydroxyalkanoates (PHA). Both biodegradable polyesters improved the thermal properties of PHA; the flexible PBS decreased the melting point of PHB while the rigid PLA added a glass transition point to PHB. Moreover, the biodegradable polyesters lowered the crystallinity of poly(3-hydroxybutyrate) (PHB). ^1^H MAS NMR spectra at −40 °C showed broad and overlapped signals for all polymers measured. With increasing temperature, the PHA/PBS copolymer showed larger and sharper ^1^H MAS signals of the PHB unit while those of the PHA/PLA copolymer remained similarly broad to those of the original PHB. The ^13^C PST-MAS signals of the PHB unit were increased by copolymerization with PBS, in addition to the ^1^H MAS signals. Since ^1^H spin-lattice relaxation in the rotating frame (*T*_1__ρ_H) was decreased more by the copolymerization with PBS than PLA, the addition of PBS increased the amplitudes of slow molecular motions (kHz order) of the polymer chain related to the spin-locking frequency. Meanwhile, ^1^H and ^13^C spin-lattice relaxation times in the laboratory frame (*T*_1_H and *T*_1_C), which were reflected matching with rapid molecular motions (MHz order), were reduced by copolymerization not only with PBS but also with PLA. In other words, the disordered PHB chain in the amorphous moiety due to the copolymerization improved polymer compatibility between PHB and the added polyester and amplified rapid molecular motions. The compatibility enhanced the *T*_1_H relaxation such that the time reduced and approached the *T*_1_H values of the plasticized PHA. The increased rapid molecular motions of the PHB substituent promoted the *T*_1_C relaxation to shorten the *T*_1_C value of CH_2_ in the plasticized PHA. Our present study makes it possible to use solid-state NMR parameters as the index of molecular mobility and polymer compatibility. Studies of production process and mechanical properties using other biomass-based polymers using the solid-state NMR parameters are currently in progress.
